# Microglial activation occurs in the absence of anxiety-like behavior following microembolic stroke in female, but not male, rats

**DOI:** 10.1186/s12974-014-0174-7

**Published:** 2014-11-06

**Authors:** Christina L Nemeth, Renuka Reddy, Mandakh Bekhbat, Jabari Bailey, Gretchen N Neigh

**Affiliations:** Department of Psychiatry and Behavioral Science, Emory University, Atlanta, GA USA; Department of Physiology, Emory University, Atlanta, GA USA

**Keywords:** Sex differences, Vascular ischemia, Animal model, Behavior, Microglia

## Abstract

**Background:**

The incidence of depression and anxiety disorders is twice as high in women than men; however, females exhibit less neuronal damage following an equivalent ischemic event. Microembolic stroke increases anxiety- and depressive-like behaviors in male rats but the behavioral repercussions in females are unknown.

**Findings:**

Given the relative neuronal protection from stroke in ovary-intact females, female rats exposed to microembolic stroke may be behaviorally protected as compared to males. The data presented demonstrate that anxiety-like behavior is increased in males despite a comparable increase in microglial activation following microembolic stroke in both males and females.

**Conclusions:**

These data suggest that males may be more behaviorally susceptible to the effects of microembolic stroke and further illustrate a dissociation between neuroinflammation and behavior in females.

## Findings

Though it is unclear whether men and women differ in the presentation of microvascular lesions, women experience reduced incidence of coronary heart disease and stroke [1,2]. Female sex steroids have been shown to garner protection following ischemia putting premenopausal women at an advantage over men with regards to recovery [3]. In cases of traumatic brain injury, women experience reduced secondary edema and better functional outcome compared to men, a finding consistent with rodent models of injury [4]. However, females report a higher incidence of depression and anxiety following stroke [1], and cerebrovascular disease appears to be more depressogenic among women than men [5].

The variable results regarding the influence of sex on outcome from ischemia do not allow for clear generalizations, but indicate that effects observed in males cannot be reliably extrapolated to females. Previous findings show that the induction of microembolic infarcts induces behavioral disruption after long-term recovery in male rats [6] and, therefore, the current study builds upon these findings to compare the behavioral and histological effects of microembolic stroke between males and females. Here, we used the same microsphere embolism (ME) model [6] to measure the behavioral and microglial response in both male and female rats to determine whether ME-induced cerebral modifications were consistent between male and female rats. We chose to focus on microglial activation in parallel with anxiety-like behavior because of previous work demonstrating a role of microglial activation in anxiety-like behavior [7-9].

## Materials and methods

Adult male and female Wistar rats (3 months of age, Charles River, Wilmington, MA, USA) were pair-housed by sex until surgery. An AAALAC-approved facility maintained the rats on a reverse 14:10 light:dark cycle in a temperature- and humidity-controlled vivarium with *ad libitum* food and water. We performed all experiments in accordance with the Institutional Animal Care and Use Committee of Emory University and the National Institutes of Health *Guide for the Care and Use of Laboratory Animals*. Following at least a 1-week acclimation period, rats were randomly assigned to sham (male n = 10; female n = 10) or microsphere embolism (ME; male n = 10; female n = 11) surgical groups. For ME surgeries, rats were isoflurane anesthetized and secured in a supine position. A neck incision was made and the common carotid artery was isolated and ligated followed by suture ligation of the external carotid at the bifurcation with the internal carotid artery. Microspheres (New England Nuclear Inc., Boston, MA, USA; 50 μm in diameter; suspended in 10% dextran and 0.01% Tween in isotonic saline; approximately 2,500 spheres in 50 μl) were injected, using a 30-G needle, into the left internal carotid artery. Female estrous cycle stages were tracked by vaginal lavage prior to surgery and surgeries were performed during diestrus.

**Figure 1:**
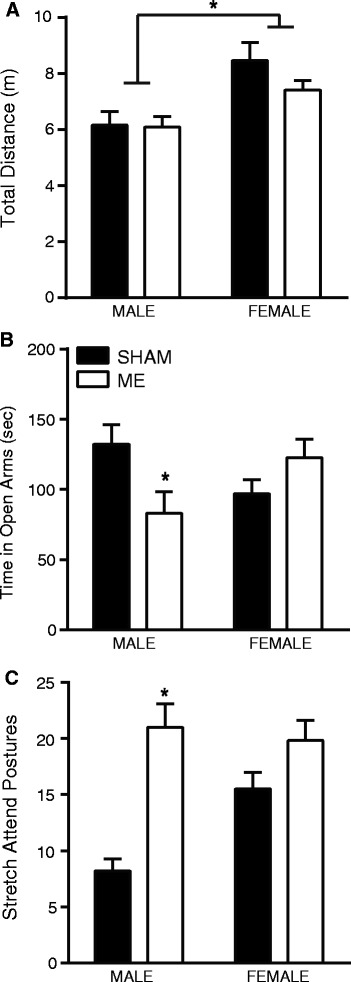
**Male and female sham and microsphere embolism (ME) rats were run in a 5-minute elevated plus maze. (A)** Total distance traveled of male and female sham and ME rats indicated a significant effect of sex, such that female rats traveled more overall as compared to male rats (*P* <0.05). **(B)** A significant interaction between sex and surgery was detected in the elevated plus maze: specifically, compared to male sham, male ME rats spent less time in the open arms of the elevated plus maze, indicative of an anxiety-like state. No difference was detected between female sham and ME rats (*P* <0.05). **(C)** Similarly, the number of stretch attend postures were significantly higher in male ME rats compared to both sham and female rats (*P* <0.05). For all, error bars represent standard error of the mean (SEM) and *indicates *P* <0.05.

The elevated plus maze (EPM) was used as a measure of anxiety-like behavior which consisted of a 5-minute exposure on Day 14 during the animals’ dark cycle. While general locomotor behavior did not differ as a result of ME (*P* >0.05; Figure 1A), females (sham and ME) were more active in the EPM compared to male rats (F_1,36_ = 14.92, *P* <0.05). Furthermore, analysis of time in the open arms of the EPM revealed a significant interaction of sex and surgery, such that ME procedures affected male, but not female, behavior at 2 weeks (F_1,43_ = 7.140, *P* <0.05; Figure 1B). Bonferroni posttests revealed a difference between male sham and male ME time spent in the open arms of the maze (mean difference: 49.03 s, *P* <0.05). Females that underwent the ME procedure did not demonstrate any evidence of increased anxiety-like behavior as defined by differences in time spent in the open arms of the maze, or in stretch attend postures. Conversely, males had an increased display of stretch attend postures compared to sham-operated male and female rats (interaction: F_1,42_ = 7.004, *P* <0.05; posttest: mean difference: 12.80, *P* <0.01; Figure 1C).

Twenty-four hours following EPM behavior, rats were transcardially perfused with 4% paraformaldehyde and brains were removed and stored at 4°C until sectioned at 40 μM. Sections (section sampling frequency = 12) were taken for IBA1 (hippocampus, caudate nucleus, amygdala) immunohistochemistry and cell morphology. IBA1 (rabbit anti-IBA1, 1:500; Wako, Richmond, VA, USA) sections were stained and analyzed as previously described [6]. IBA1 count data in the hippocampus, amygdala, and caudate nucleus are expressed as the estimated number of cells. Cell morphology in the hippocampus was assessed from the same sections in which 25 to 30 cells were chosen at random, converted to 8-bit, cleaned with a Gaussian filter, binarized, and analyzed for the number of branches, the number of junctions, and average branch length using the ImageJ AnalyzeSkeleton plugin (National Institutes of Health, version 1.47). For all analyses, the investigator was blind to treatment group.

**Figure 2:**
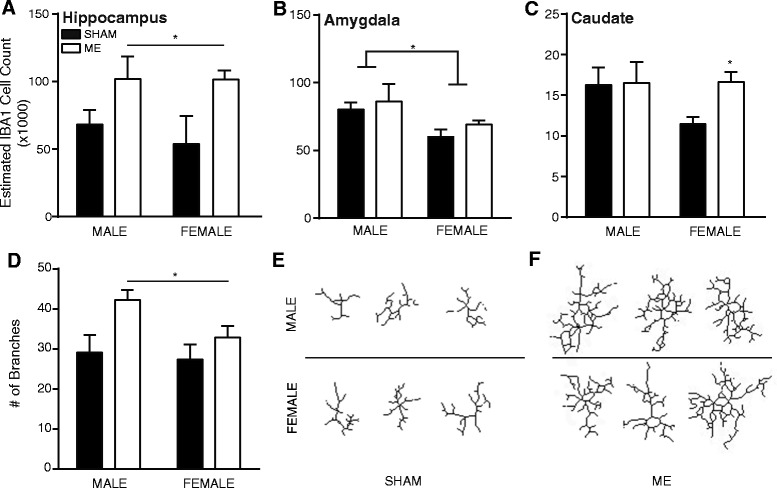
**In depth microglia/macrophage activation was determined by cellular counts of ionized calcium-binding adapter molecule 1 (IBA1)-stained cells in the brains of male and female sham and ME rats. (A)** IBA1 cell counts were estimated in the hippocampus of male and female sham and ME rats and compared with a 2-way ANOVA. Analysis revealed a main effect of surgery such that ME procedures increased the number of activated microglial cells independent of sex. **(B)** In contrast, staining in the amygdala revealed an effect of sex, with females having fewer overall IBA1+ cells as compared to males (*P* <0.05). **(C)** *A priori t*-test of the estimated cell counts in the caudate nucleus showed and increased number of stained cells (*P* <0.05); through higher at baseline, males showed no effect of ME. **(D)** Morphological assessment of IBA1+ cells in the hippocampus illustrated increased branching and a hyper-ramified state in both male and female ME rats compared to sex-matched sham animals (*P* <0.05). Representative cells for male and female sham **(E)** and ME rats **(F)** are shown. For all, error bars represent standard error of the mean (SEM) and *indicates *P* <0.05.

A 2-way ANOVA confirms that in the hippocampus, both male and female rats showed increased estimated counts of IBA1+ cells following ME surgery (main effect of surgery: F_1,14_ = 8.018, *P* <0.05; Figure 2A). Conversely, a main effect of sex was present in the amygdala (F_1,11_ = 7.68, *P* <0.05; Figure 2B) indicating an overall decreased microglial detection in both sham and ME female rats. Though no differences were detected via a 2-way ANOVA in the caudate nucleus, separate examination of male and female rats using *a priori* student’s *t*-test revealed an increase in female IBA1 cells following ME and suggests that the cellular response in the caudate nucleus may be more sensitive in females compared to males (t(6) = 3.40, *P* <0.05; Figure 2C).

A 2-way ANOVA of the morphology of IBA1+ cells in the hippocampus showed no effect of sex; however, ME surgery significantly increased both the number of branches (F_1,12_ = 7.73, *P* <0.05; Figure 2D) as well as the number of junctions (F_1,12_ = 7.57, *P* <0.05) without affecting the average branch length (*P* >0.05). Representative cells are depicted for sham and ME rats in Figures 2E and 2F, respectively.

## Conclusions

Collectively, these data demonstrate that males are more susceptible than females to the behavioral effects of ME and suggest that microglial activation is brain region specific and is not coupled to anxiety-like behavior in female rats. In regard to the males, the data extend previous documentation of the behavioral effects of ME to include an additional metric of anxiety-like behavior, the elevated plus maze. Though our previous studies document anhedonia, social and anxiety-like dysfunctions, in addition to the effects of ME on lesion volume and the general presence of microglia [6], our current work refines our understanding of the histological effect of ME by documenting an increase in reactive microglia and an altered morphology of those cells. A causal relationship between microglial activation and anxiety-like behavior has been demonstrated following global ischemia [7] and these data suggest that a similar relationship may exist following ME in male rats.

The absence of altered behavior following ME infarction in females, despite clear evidence of microglial activation, may illustrate a resilience that stems from the neuroprotective effects of endogenous estrogen. Though we detected no behavioral abnormalities in females following ME, it is possible that behavioral deficits exist in other metrics of behavior not measured here, and that these deficits are linked to inflammation within the caudate nucleus and/or hippocampus. Clinical and rodent literature support the role of estrogen as a neuroprotectant following stroke or other brain trauma [3,10] and studies of other rodent ischemia models note reduced infarct size in female rodents compared to both males and ovariectomized female counterparts [11] (thoroughly reviewed in [2]), though no studies have examined the long-term effects of microvascular injury in male and female rats. We have previously reported that frank lesions are rare following ME procedure in male rats [6] and, in the current study, we demonstrate that microglial activation is region dependent, and that microglia within the hippocampus adopt a hyper-ramified state. The enhanced ramification, illustrated by increased branching and junctions observed in these cells, is likely a heightened response to ME that stems from systemic inflammation and serves to protect the damaged area from additional injury [12-14]. Importantly, these specific cellular adaptations do not associate with behavior suggesting that the mechanism for the behavioral difference within the current examination is not at the level of neuronal damage. The possibility remains that female sex steroids may be modifying the functional consequences of ME-induced microglial activation as has been reported for chronic stress [15]. Interactions among estrogen and microglia are diverse and plentiful [15], and estrogen has been shown to simultaneously stimulate microglia and neural repair [16]. Alternatively, sex differences in neuronal or signaling reorganization and plasticity unrelated to microglia/macrophage activation may mediate these differences in behavior [17].

Collectively, these data demonstrate that following microembolic stroke, both male and female rats exhibit evidence of neuroinflammation with increased numbers of ‘primed’ microglial cells, but that females do not exhibit corresponding anxiety-like behaviors. A relationship between anxiety-like behavior and neuroinflammation has been previously demonstrated in male rats in models of global ischemia [7] and chronic stress [8,9]. The current data set suggests that this relationship cannot be extrapolated to the female brain. Future studies investigating the mechanisms by which females exhibit normal behavior in the presence of microglial activation will provide important insight for treatment strategies.

## Abbreviations

ME: microsphere embolism
EPM: elevated plus maze
IBA1: ionized calcium-binding adapter molecule 1
